# Danger or Salvation? The Role of DAMPs in Cancer Therapy

**DOI:** 10.3390/cancers18091442

**Published:** 2026-04-30

**Authors:** Anna A. Vedunova, Evgenii L. Guryev, Sergey V. Gudkov, Tatiana A. Mishchenko, Maria V. Vedunova

**Affiliations:** 1 Institute of Biology and Biomedicine, Lobachevsky State University of Nizhny Novgorod, 23 Gagarin Ave., 603022 Nizhny Novgorod, Russia; anna.vedunova@unn.ru (A.A.V.); eguryev@ibbm.unn.ru (E.L.G.); saharnova87@mail.ru (T.A.M.); mvedunova@yandex.ru (M.V.V.); 2 Prokhorov General Physics Institute of the Russian Academy of Sciences, Vavilov Str. 38, 119991 Moscow, Russia; 3 Department of Fundamental Sciences, Bauman Moscow State Technical University, 5 2nd Baumanskaya St., 105005 Moscow, Russia

**Keywords:** cancer, cancer immunotherapy, regulated cell death, immunogenic cell death, damage-associated molecular patterns

## Abstract

**Background:** Modern oncology views immune system dysfunction as a key factor in carcinogenesis. The induction of immunogenic cell death (ICD), a form of regulated cell death capable of activating adaptive immunity, represents a promising therapeutic strategy. Damage-associated molecular patterns (DAMPs) play a central role in this process. This review aims to summarize current knowledge of DAMPs, their release mechanisms during ICD, their classification, and their prognostic and therapeutic significance in antitumor immunity. **Methods:** We systematically reviewed and synthesized literature published in Pubmed and Google Scholar on ICD and DAMPs, focusing on distinct forms of DAMPs which were categorized based on recognition mechanisms (five classes) and cellular origin (extracellular, mitochondrial, nuclear, and cytosolic). Key molecules, their receptors, downstream signaling pathways, and clinical associations were analyzed. **Results:** The spatiotemporally coordinated release of the pattern of DAMPs promotes dendritic cell maturation, antigen presentation, activation of cytotoxic T lymphocytes, and elimination of tumor cells. DAMPs can exhibit a dual role: they are able to induce sterile inflammation essential for antitumor immunity, but may also contribute to metastasis and chronic inflammation. Among all DAMPs, high-mobility group box 1 (HMGB1, a nuclear DAMP) and calreticulin (CRT, a cytosolic protein) demonstrate the greatest prognostic value. Other DAMPs (e.g., extracellular matrix components, uric acid) act as signal amplifiers during various forms of cell death. **Conclusions:** Understanding the spatiotemporal dynamics of DAMP release is critical for activating immune responses against malignant cells. Monitoring DAMPs may improve patient stratification, predict therapeutic responses, and enable personalized immunotherapeutic strategies. Further investigation of ICD mechanisms and DAMP release represents a fundamental basis for developing novel anticancer therapies.

## Simple Summary

The immune system plays a crucial role in fighting cancer. One promising approach to cancer treatment involves inducing a specific type of cell death termed immunogenic cell death (ICD). When cancer cells are dying through ICD, they release special alarm signals known as damage-associated molecular patterns (DAMPs). These signals include molecules such as calreticulin, high-mobility group box 1 (HMGB1), and adenosine triphosphate (ATP). DAMPs alert the immune system, recruit dendritic cells, and activate cytotoxic T lymphocytes, which then recognize and destroy remaining tumor cells. However, DAMPs have a dual role: while they help eliminate cancer, they can also promote inflammation that may support tumor spread. This review examines the different types of DAMPs, how they are released, and their effects on antitumor immunity. Understanding the coordinated release of DAMPs may help predict which patients will respond to therapy and guide the development of novel anticancer treatment modes.

## 1. Introduction

According to current concepts, cancer development is, to varying degrees, associated with dysfunction of the immune system and impaired immunological surveillance of oncogenic viruses or transformed cells [1,2]. Understanding the role of the immune system in tumorigenesis has provided the foundation for the development of various immunotherapeutic strategies aimed at inhibiting tumor growth and stimulating antitumor immune responses [3]. Immunotherapy is based on overcoming immune suppression, promoting recognition of tumor cells by the immune system, and restoring the body’s capacity to mount an effective antitumor response [4,5].

Among contemporary approaches, particular attention is given to immunotherapeutic strategies that induce various forms of regulated cell death that possess a strong immunogenic effect. Regulated cell death, defined as a genetically controlled process, has long been considered immunologically ‘silent’ or even tolerogenic [6]. However, accumulating evidence indicates that, under certain conditions, stress-induced cell death can elicit an inflammatory response that leads to activation of adaptive immunity and the formation of long-term immunological memory. This functionally distinct form of cell death has been termed immunogenic cell death (ICD) [7].

ICD is an umbrella term encompassing several forms of regulated cell death, including immunogenic forms of apoptosis (i.e., immunogenic apoptosis), necroptosis (i.e., immunogenic necrosis), pyroptosis, and ferroptosis [7,8,9,10]. Emerging evidence shows that a recently described copper-dependent form of regulated cell death, termed cuproptosis [11,12], as well as a convergent form of pyroptosis, apoptosis and necroptosis, termed PANoptosis [13,14,15], and paraptosis [16,17,18] also have promising immunogenic potential. A common hallmark of these processes is the release of damage-associated molecular patterns (DAMPs) and/or cytokines and chemokines, which induce robust antitumor immune responses [8,19]. The term “damage-associated molecular patterns” was introduced following the formulation of the “danger model” proposed by Polly Matzinger in 1994 [20]. DAMPs are endogenous molecules released or exposed by cells in response to various forms of damage, including injury, ischemia, oncogenic transformation, and certain therapeutic interventions. Because DAMP-induced inflammatory responses occur independently of infectious agents, they are referred to as sterile inflammation [21,22]. DAMPs exhibit a dual role: intracellularly, they participate in normal cellular processes, whereas upon release into the extracellular space, they act as danger signals that activate the immune system [23,24]. According to this concept, the key triggers of sterile inflammation are danger signals—alarmins—released from damaged or dying cells [20,23,25]. Heat shock proteins (HSPs) were among the first molecules identified as DAMPs [26,27].

This review summarizes the current understanding of DAMPs and highlights key representatives that are of particular importance for the development of modern therapeutic approaches.

## 2. The Concept of DAMPs and Their Role in the Body

The innate immune system is capable of recognizing “foreign” molecular structures derived from pathogens, referred to as pathogen-associated molecular patterns (PAMPs), through specialized receptors known as pattern recognition receptors (PRRs). These receptors are expressed on virtually all immune cells, including monocytes, macrophages, dendritic cells (DCs), neutrophils, mast cells, natural killer (NK) cells, eosinophils, and T and B lymphocytes, as well as on non-immune barrier cells such as endothelial and epithelial cells and fibroblasts [21,28]. Similar to pathogen-induced inflammation, DAMPs can activate both non-immune and innate immune cells, including neutrophils, macrophages, and DCs. Activation of these cells leads to the production of cytokines and chemokines, which subsequently recruit lymphocytes and initiate adaptive immune responses [8,23,25]. In addition, certain DAMPs are capable of directly activating cells of the adaptive immune system [22].

Recognition of DAMPs induces a specific immune response that plays a crucial role in antitumor immunity and tissue regeneration. At the same time, it may also contribute to the development of various inflammatory diseases, including metabolic disorders, neurodegenerative conditions, autoimmune diseases, and cancer metastasis [29,30]. Therefore, a balance between activation and regulation of innate immunity is essential for maintaining homeostasis.

### 2.1. Mechanisms of DAMP Release During Immunogenic Cell Death (ICD)

**Figure 1:**
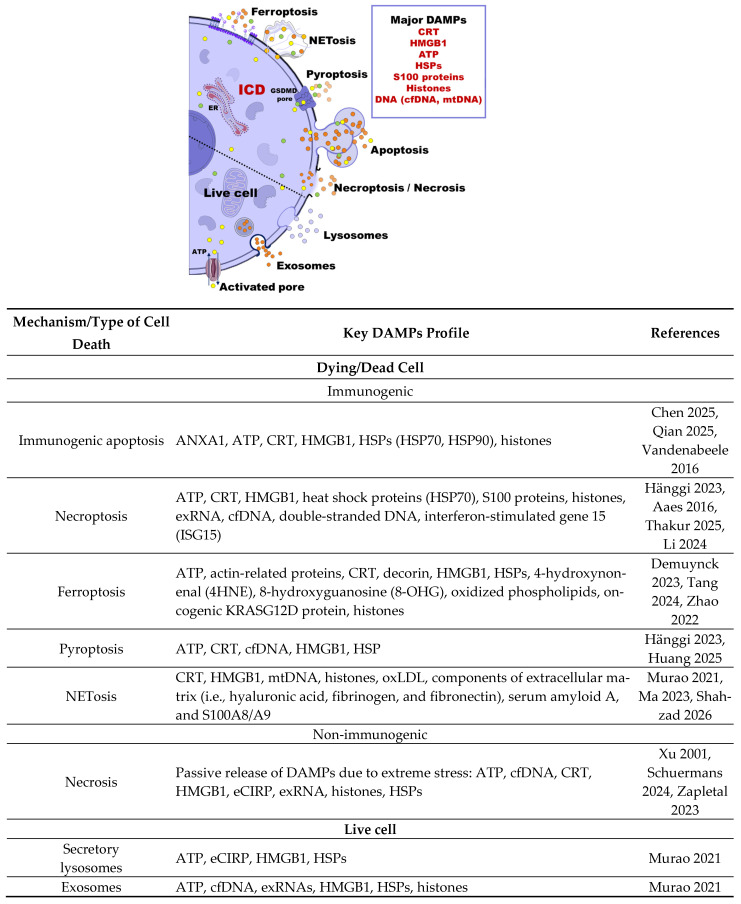
Pathways of damage-associated molecular patterns (DAMPs) release during different types of immunogenic cell death (ICD). The scheme is divided along a central axis into lytic pathways, which involve disruption of the plasma membrane, and vesicular pathways, which preserve the membrane barrier. Lytic pathways are characterized by the passive (i.e., non-immunogenic necrosis) or rapid (i.e., immunogenic necroptosis, pyroptosis, and ferroptosis) release of intracellular contents. Pyroptosis represents a distinct case of the classical pathway of gasdermin D (GSDMD) activation, in which pore formation in the plasma membrane serves as a critical mechanism of controlled rupture. In vesicular and secretory pathways (i.e., apoptosis, exosomes, secretory lysosomes), DAMPs are released in a compartmentalized manner, allowing signals to be transmitted over a distance without generating a focus of sterile necrotic inflammation. NETosis represents a hybrid mechanism, combining nuclear envelope disintegration with the release of chromatin fibers decorated with proteases to form specific neutrophil extracellular traps (NETs). Abbreviations: ATP—adenosine triphosphate, cfDNA—circulating free DNA, CRT—calreticulin, DAMPs—damage-associated molecular patterns, eCIRP—extracellular cold-inducible RNA-binding protein, ER—endoplasmic reticulum, exRNAs—extracellular RNAs, HMGB1—high-mobility group box 1, HSPs—heat shock proteins, ICD—immunogenic cell death, mtDNA—mitochondrial DNA, NET—neutrophil extracellular traps, oxLD—oxidized low-density lipoprotein. References: immunogenic apoptosis [34,35,36], necroptosis [6,37,38,39], ferroptosis [9,40,41], pyroptosis [6,42], NETosis [31,43,44], Necrosis [45,46,47], secretory lysosomes [31], exosomes [31].

During ICD, tumor cells expose DAMPs such as calreticulin (CRT) and other chaperones on their surface, release high-mobility group box 1 (HMGB1), secrete adenosine triphosphate (ATP), and induce type I interferon (IFN I) response, which promotes the production of T-cell-attracting cytokines [8,31] (Figure 1).

The mechanisms of DAMP release vary depending on the molecular type. DAMPs may be passively released following membrane rupture during necrosis (non-immunogenic) or necroptosis, or actively and energy-dependently, as observed in apoptosis, pyroptosis, ferroptosis, and NETosis (Figure 1). In addition, some DAMPs can be actively secreted by viable cells through exocytosis of secretory lysosomes or exosomes, as well as through the activation of membrane pores [31,32] (Figure 1).

The spatiotemporally coordinated pattern of DAMP release enhances phagocytic activity and promotes DC maturation, facilitating the clearance of dying cancer cells and their debris. This process supports DC migration to lymph nodes and the initiation of a cytotoxic T-lymphocyte response involving both αβ and γδ T cells. Such a robust tumor-specific T-cell response is often sufficient to eliminate cancer cells that survive the initial cytotoxic stimulus [7,8,33].

**Figure 2:**
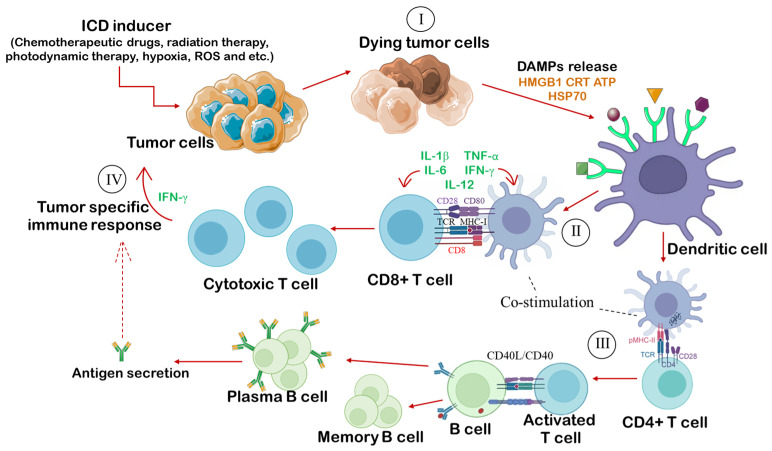
Scheme of immunogenic cell death (ICD) induction. The scheme outlines the four stages of the adaptive immune response initiated by tumor cells undergoing ICD and its coordination with innate immunity for tumor elimination. (I) Induction of ICD and DAMP release: ICD inducers trigger dying tumor cells to release the pattern of DAMPs, including HSP70, CRT, HMGB1, and ATP, along with chemokines such as CXCL1, CCL2, and CXCL10, thereby mimicking an infectious process; (II) Antigen uptake and DC maturation: DAMPs and tumor antigens promote the phagocytosis of dying cells by DCs. DCs process antigens and present them via MHC I and MHC II, express CD80, mature, and migrate to the lymph nodes; (III) T-cell polarization and proliferation: Mature DCs activate CD4^+^ and CD8^+^ T lymphocytes. In the presence of IL-12 and IFNγ, CD4^+^ cells polarize toward a Th1 phenotype, while CD8^+^ cytotoxic T lymphocytes proliferate; (IV) Effector phase: Activated IFNγ-producing CD8^+^ T cells induce an antitumor cytotoxic response, eliminating both the primary lesion and resistant tumor cells. Abbreviations: ATP—adenosine triphosphate, CCL2—C-C motif chemokine ligand 2, CD—cluster of differentiation, CXCL1—C-X-C motif chemokine ligand 1, CXCL10—C-X-C motif chemokine ligand 10, CRT—calreticulin, CTL—cytotoxic T lymphocyte, DAMPs—damage-associated molecular patterns, DC—dendritic cells, HMGB1—high-mobility group box 1, HSP70—heat shock protein 70, ICD—immunogenic cell death, IFNγ—interferon gamma, IL-12—interleukin 12, MHC—major histocompatibility complex, Th1—T helper 1.

Furthermore, tumor cells undergoing ICD exhibit features of “modified self-mimicking”, characterized by a chemokine release profile resembling that of infectious agents, including the simultaneous secretion of C-X-C motif chemokine ligand 1 (CXCL1), C-C motif chemokine ligand 2 (CCL2), and C-X-C motif chemokine ligand 10 (CXCL10) [7,8,48] (Figure 2). This chemokine combination preferentially recruits neutrophils, which represent the first line of innate immune defense and contribute to the elimination of residual tumor cells through respiratory burst mechanisms, including the production of reactive oxygen species (ROS) and nitric oxide [49].

### 2.2. Therapeutic Modulation of DAMP-Driven Antitumor Immunity

Different modalities of conventional anticancer therapy exert distinct effects on DAMPs production by tumor cells. Surgical intervention, by inducing systemic inflammation and transient immunosuppression, may in some cases facilitate tumor cell dissemination. Certain anesthetic agents have also been reported to suppress immune function in the postoperative period, which is considered an unfavorable prognostic factor associated with metastasis [50,51]. In contrast, DAMPs actively released by apoptotic cells play a beneficial role in anticancer therapy through their interaction with the immune system. Both chemotherapy and radiotherapy can induce immunogenic apoptosis [23,52].

Exposure of tumor cells to ionizing radiation leads to the generation of ROS and endoplasmic reticulum (ER) stress, which are essential for DAMP release. DAMPs, including HMGB1 and ATP, as well as CRT and HSPs, recruit antigen-presenting cells (such as macrophages and DCs) via chemokine-mediated mechanisms to mount an antitumor response [53].

Activation of antigen-presenting cells depends on the interaction of DAMPs with PRRs, including purinergic receptor P2X7 (P2RX7), purinergic receptor P2X2 (P2RY2), cluster of differentiation 91 (CD91), cluster of differentiation 40 (CD40), and toll-like receptor 4 (TLR4). P2RX7 promotes the secretion of interleukin 18 (IL-18) and interleukin-1 beta (IL-1β) via the NLR family pyrin domain containing 3/Apoptosis-associated speck-like protein containing a CARD/caspase-1 (NLRP3/ASC/caspase-1) pathway, whereas the interaction of ATP with P2RY2 facilitates the recruitment of immature DCs. Engagement of CRT with CD91 induces the release of tumor necrosis factor alpha (TNF-α) and interleukin-6 (IL-6); CD91 also serves as a receptor for HSP90. Binding of HSP70 to CD40 activates CD8^+^ cytotoxic T lymphocytes, while interaction of HMGB1 with TLR4 stimulates the production of proinflammatory cytokines. Through interactions with PRRs, DAMPs activate DCs, which subsequently engulf tumor cells, process and present tumor antigens via major histocompatibility complex class I (MHC I) molecules, and migrate to lymph nodes, where they promote the differentiation of T cells into the CD8^+^ phenotype. These CD8^+^ T cells are then activated and differentiate into cytotoxic T lymphocytes (CTLs), which induce tumor cell apoptosis through the release of perforin and granzyme B or via activation of the Fas ligand/Fas receptor (FasL/FasR) pathway [54,55].

Thus, DAMPs represent a double-edged signaling system: their controlled release during ICD triggers protective antitumor immunity, whereas their dysregulation promotes chronic inflammation and disease progression. Harnessing DAMP-driven immunogenicity through radiotherapy or chemotherapy or induction of novel ICD-inducing agents offers therapeutic promise, but requires maintaining homeostatic balance between immune activation and resolution.

## 3. Types of DAMPs

### 3.1. Classification of DAMPs in the Context of Sterile Inflammation

**Figure 3:**
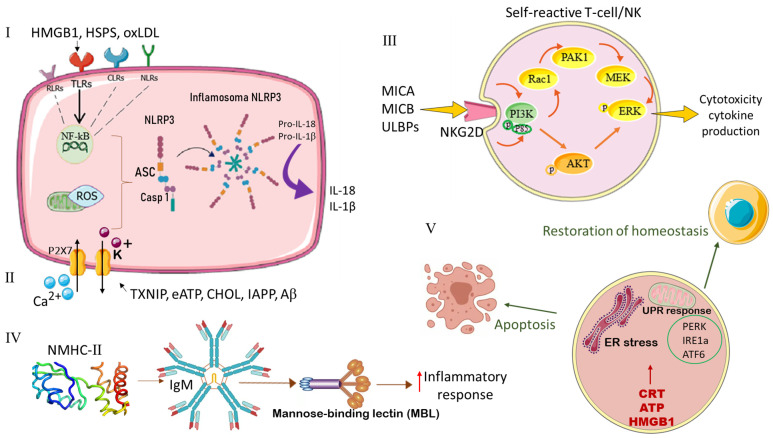
Classification of DAMPs according to their ability to activate dendritic cells. Class I: HMGB1, HSPs, oxLDL. Recognized by TLRs, CLRs, NLRs, RLRs. Initiate activation of the NLRP3 inflammasome, production of IL-1β and IL-18. Inflammasomes regulate pyroptosis and inflammation, require ASC for caspase-1 activation; Class II: TXNIP, eATP, cholesterol, amylin/IAPP, amyloid β. Directly activate NLRP3, releasing IL-1β and IL-18. Associated with ROS formation. eATP acts as a “hybrid DAMP”, activating NLRP3 via P2X7 and pannexin-1, causing changes in cellular ionic composition; Class III: MICA, MICB, ULBPs (MHC-associated proteins). Recognized by NKG2D on NK and γδ T cells. Initiate a cytotoxic response against stressed cells; Class IV: NMHC-IIA, actin, oxidized phospholipids. They are neoepitopes arising from oxidative damage. Activate PRRs, including TLRs and scavenger receptors, as well as pentraxins and complement. OSEs, such as NMHC-II, activate autoreactive IgM, which trigger MBL-mediated complement; Class V: Homeostatic DAMPs, reflecting disruptions in intracellular/extracellular homeostasis associated with ER stress. Recognized by UPR sensor molecules: PERK, IRE1α, ATF6. Such DAMPs include translocation of CRT to the cell surface, secretion of ATP, and release of HMGB1 (associated with ICD). Abbreviations: ASC—apoptosis-associated speck-like protein containing a CARD, ATF6—activating transcription factor 6, CLRs—C-type lectin receptors, CRT—calreticulin, DAMPs—damage-associated molecular patterns, eATP—extracellular adenosine triphosphate, ER—endoplasmic reticulum, HMGB1—high-mobility group box 1, HSPs—heat shock proteins, ICD—immunogenic cell death, IL—interleukin, IRE1α—inositol-requiring enzyme 1α, MBL—mannose-binding lectin, MICA—MHC class I polypeptide-related sequence A, MICA—MHC class I polypeptide-related sequence B, NK—natural killer; NKG2D—natural killer group 2 member D; NLRs—NOD-like receptors, NLRP3—NLR family pyrin domain containing 3, NMHC-IIA—non-muscle myosin heavy chain IIA, oxLDL—oxidized low-density lipoproteins, OSEs—oxidative-specific epitope, P2X7—purinergic receptor 7; PERK—RNA-like endoplasmic reticulum kinase, RLRs—RIG-I-like receptors, ROS—reactive oxygen species; TLRs—toll-like receptors, TXNIP—thioredoxin-interacting protein, ULBPs—UL16-binding proteins, UPR—unfolded protein response.

In the context of sterile inflammation, DAMPs are classified according to their recognition mechanisms into five classes [24] (Figure 3).

#### 3.1.1. Class I DAMPs

Class I DAMPs, including HMGB1, HSPs, and oxidized low-density lipoproteins (oxLDL), act as potent danger signals that are predominantly recognized by toll-like receptors (TLRs). This recognition initiates a cascade leading to activation of the NLRP3 inflammasome and subsequent production of the proinflammatory cytokine interleukin-1β (IL-1β) [28]. However, recognition of class I DAMPs is not restricted to TLRs. C-type lectin receptors (CLRs), nucleotide-binding oligomerization domain (NOD)-like receptors (NLRs), and RIG-I-like receptors (RLRs) also participate in their detection, thereby broadening the spectrum of immune responses [56,57].

Among class I DAMPs recognized by CD91, CRT deserves particular attention. CRT is an endoplasmic reticulum chaperone that, upon translocation outside the ER, assumes multiple important functions on the cell surface and in the extracellular environment (Figure 3).

#### 3.1.2. Class II DAMPs

Class II DAMPs, including thioredoxin-binding protein (TXNIP), extracellular adenosine triphosphate (eATP), cholesterol, amylin (i.e., islet amyloid polypeptide, IAPP), and amyloid β, are capable of directly activating the NLRP3 inflammasome, thereby initiating the release of proinflammatory cytokines, such as IL-1β and IL-18 [58,59]. Importantly, activation of the NLRP3 inflammasome is closely associated with the generation of intracellular ROS, highlighting its significant role in this process [60]. Among DAMPs, eATP is distinguished by its unique properties and may be considered a “hybrid DAMP”, mediating both direct and indirect mechanisms of NLRP3 activation. Specifically, eATP induces canonical activation of the NLRP3 inflammasome through interaction with P2X7 receptors and pannexin-1 hemichannels. Initial activation of P2X7 results in alterations in intracellular ionic homeostasis, including calcium (Ca^2+^) influx and potassium (K^+^) efflux, thereby triggering a cascade of downstream events leading to inflammasome activation [61,62] (Figure 3).

#### 3.1.3. Class III DAMPs

Class III DAMPs, such as MHC-associated proteins including MHC class I polypeptide-related sequence A (MICA), MHC class I polypeptide-related sequence B (MICB), and UL16-binding proteins (ULBPs), are recognized by the natural killer group 2 member D (NKG2D) receptor expressed on the surface of NK cells and γδ T cells [28]. Interaction between class III DAMPs and NKG2D initiates signaling cascades that activate cytotoxic responses directed against cells exhibiting signs of stress or damage [58,63]. This mechanism plays a critical role in immune surveillance and the maintenance of tissue homeostasis [60] (Figure 3).

#### 3.1.4. Class IV DAMPs

Class IV DAMPs, such as non-muscle myosin heavy chain IIA (NMHC-IIA) neoantigens, cytoskeletal actin, and oxidized phospholipids, typically represent neoepitopes that arise as a result of oxidative damage [28]. These oxidative-specific epitopes (OSEs) constitute a conserved set of structures present on various oxidatively modified self-proteins and lipids [58,64]. Under conditions of pathological stress due to excessive ROS production, OSEs accumulate and bind to PRRs, thereby initiating sterile inflammation [60]. A classic example of this process is ischemia–reperfusion injury [65]. OSEs have been shown to interact with a wide range of recognition receptors, including TLRs and scavenger receptors expressed by macrophages, as well as soluble components of innate humoral immunity, such as pentraxins and complement proteins [66]. Experimental studies in intestinal and cardiac ischemia–reperfusion models have demonstrated that OSEs, including NMHC-II, activate autoreactive natural immunoglobulin M (IgM) antibodies, which in turn trigger the mannose-binding lectin (MBL)--mediated complement activation cascade, thereby amplifying the inflammatory response [67,68] (Figure 3).

#### 3.1.5. Class V DAMPs

Class V DAMP signals, from the perspective of homeostatic danger signaling, have recently been proposed as a distinct class of DAMPs that reflect disturbances in the stable intracellular and/or extracellular microenvironment. These homeostatic DAMPs associated with ER stress are recognized by three key unfolded protein response (UPR) sensor molecules: protein kinase RNA-like endoplasmic reticulum kinase (PERK), inositol-requiring enzyme 1α (IRE1α), and activating transcription factor 6 (ATF6) [69,70,71].

Class V DAMPs are generated under conditions of disrupted intracellular homeostasis that induce ER stress and activation of the UPR. Such conditions include the accumulation of misfolded or oxidized proteins, alterations in pH or osmolarity, and hypoxia [67]. These perturbations are detected by UPR sensor molecules (PERK, IRE1α, ATF6), which initiate adaptive responses aimed at restoring cellular homeostasis or, in the case of irreversible damage, trigger cell death programs such as apoptosis or necrosis [72]. Among the most significant DAMPs associated with ICD and influencing the clinical response of tumor cells are the translocation and surface exposure of CRT during the pre-apoptotic phase, the secretion of ATP into the extracellular space, and the post-apoptotic release of HMGB1 into the tumor microenvironment [7,23] (Figure 3).

All classes of DAMPs are capable of promoting DC maturation either through direct activation of DC signaling pathways or through the induction of other cellular and humoral innate immune processes that indirectly facilitate DC maturation [73].

An alternative classification of DAMPs is based on their cellular origin and the mechanisms by which they are released during cell death.

### 3.2. Classification of DAMPs by Cellular Origin and Release Mechanisms

#### 3.2.1. Extracellular DAMPs

Tissue injury leads to degradation of the extracellular matrix, particularly in loose fibrous connective tissue, resulting in the release of extracellular DAMPs. This process generates fragments of extracellular matrix components, including hyaluronic acid, heparan sulfate, and biglycan, which arise through proteolysis mediated by enzymes released from dying cells or by proteases activated during tissue repair and remodeling [74]. In addition, extracellular molecules such as fragments of collagen, fibronectin, and fibrinogen can function as DAMPs; these components play an important role in inflammatory processes [75].

Extracellular soluble DAMPs interact with multiple PRRs, initiating a rapid inflammatory response. DCs and macrophages activated by interleukin-1 alpha (IL-1α) and interleukin-33 (IL-33) begin de novo synthesis of additional soluble DAMPs, thereby replenishing the extracellular DAMP pool. At the same time, molecules that are normally structural components of the extracellular matrix may be proteolytically released following tissue injury and subsequently act as soluble DAMPs. The molecular composition of extracellular DAMPs is highly heterogeneous, ranging from small molecules such as uric acid or ATP to large proteins exceeding 100 kDa and even entire organelles. This structural diversity enables DAMPs to engage in cross-reactivity with PRRs as well as a broad range of non-immune receptors, thereby contributing to the complexity of DAMP-mediated signaling [76,77].

Proteoglycans (PGs) are among the best-characterized DAMPs derived from the extracellular matrix. They play a critical role in regulating innate immune responses through interactions with immunomodulatory molecules and cells, acting as DAMPs that directly activate key PRRs and downstream signaling pathways. Notably, recent studies have demonstrated that the effects of specific PGs on the inflammatory response are context-dependent and influenced by cell type and molecular structure [78]. Extracellular proteoglycans capable of activating immune responses include aggrecan, biglycan, and decorin, as well as glycoproteins such as fibronectin, fibrinogen, tenascin-C, and versican (Table 1).

Extracellular vesicles (EVs) are membrane-bound structures released by cells. Although EVs themselves are not classified as DAMPs, they participate in the energy-dependent release and transfer of danger signals. Three main types of EVs are distinguished: exosomes (approximately 30–100 nm in diameter), microvesicles (larger vesicles), and apoptotic bodies derived from dying cells. Tumor cells have been shown to increase EV release in response to therapeutic interventions, thereby contributing to tumor progression through modulation of immune and inflammatory processes. Notably, EVs can contain DAMP molecules, including HMGB1, uric acid, ATP, and HSPs. Despite their heterogeneity and the complexity of their study, EVs play an important role in cancer pathogenesis [52].

In summary, extracellular matrix-derived DAMPs, particularly proteoglycans, can trigger and sustain inflammation via multiple PRRs and non-immune receptors in a context-dependent manner influenced by cell type and molecular structure, while extracellular vesicles—though not classified as DAMPs themselves—contribute to cancer pathogenesis by transferring DAMP molecules.

#### 3.2.2. Intracellular DAMPs

Intracellular DAMPs comprise bioorganic compounds localized in the cytosol, nucleus, cell membranes, and organelles that underpin fundamental cellular functions and are released upon cellular damage. These compounds differ from extracellular DAMPs in both chemical composition and functional properties. The spectrum of PRRs responsive to intracellular DAMPs is broader. In addition to the “classical” toll-like receptors TLR2 and TLR4, it includes TLR9, the receptor for advanced glycation end-products (RAGE), interleukin-1 receptor (IL-1R), low-density lipoprotein receptor-related protein 3 (LRP3), CD91, and the cytosolic NLR receptor NLRP3, which participates in the formation of the proinflammatory NLRP3 inflammasome. The increased diversity of responsive PRRs and the range of intracellular DAMPs suggest broader pathophysiological implications of PRR-DAMP interactions in innate immune responses at stages of the inflammatory process associated with cellular damage [94,95].

##### Mitochondrial DAMPs

Most cells continuously release mitochondrial DNA (mtDNA) and proteins into extracellular vesicles. Some of these vesicles, formed within mitochondria, mediate the transfer of material between mitochondria and other organelles. Mitochondrial contents can enhance inflammation under proinflammatory conditions, although their role in the absence of inflammation remains unclear [96].

Numerous mitochondrial components and metabolic products can act as DAMPs and induce inflammation upon entry into the cytosol or extracellular environment [96] (Table 1).

Several protective mechanisms normally prevent nonspecific inflammatory responses, including autophagic recycling of permeabilized mitochondria. However, when the homeostatic capacity of these systems is exceeded or when they become dysfunctional, mitochondria-induced inflammatory responses may promote the development of autoimmune-related diseases. Furthermore, ineffective inflammatory responses induced by mitochondrial DAMPs may contribute to the development of infectious and neoplastic diseases [97].

Currently, the best-characterized mitochondria-associated DAMPs (mtDAMPs) include mtDNA, N-formyl peptides (NFP), and microRNAs associated with the regulation of mitochondrial activity.

One of the principal mechanisms initiating the immune response after mtDNA release is its interaction with TLR9. TLR9 is an innate immune receptor capable of recognizing bacteria and viruses through binding to unmethylated CpG motifs in their DNA. Upon interaction with mtDNA, TLR9 signaling is transmitted via the cytosolic adaptor protein MYD88 (myeloid differentiation primary response protein 88) to mitogen-activated protein kinase (MAPK) and the transcription factor NF-κB, thereby initiating an inflammatory response and subsequent neutrophil chemotaxis. Furthermore, the NLRP3 inflammasome, a member of the NLR family, is critically dependent on mtDNA for its activation, leading to the production of biologically active IL-1β. Evidence that mtDNA, as a molecule containing structures homologous to bacterial components, can activate innate immune responses via interaction with TLR9 has been demonstrated in multiple animal studies [98,99].

mtDNA also promotes inflammatory responses through activation of the cyclic GMP-AMP synthase-stimulator of interferon genes-interferon regulatory factor 3 (cGAS-STING-IRF3) pathway. In damaged intestinal epithelial cells, mtDNA activates macrophages in Crohn’s disease and activates the STING pathway, thereby triggering an inflammatory response. Notably, STING deficiency attenuates mtDNA-induced inflammation [79].

Several mitochondrial proteins, including mitochondrial transcription factor A (TFAM), NFP, and carbamoyl phosphate synthase 1 (CPS-1), have been described as DAMPs in both extracellular and intracellular environments. Another important group of mtDAMPs sensitive to oxidation comprises lipids such as cardiolipin [81]. Finally, certain mitochondrial metabolites, as well as mitochondrial reactive oxygen species are now considered contributors to DAMP-related responses through their accumulation and the induction of nonspecific oxidative damage to cellular proteins [98,100].

TFAM is the most abundant protein associated with mtDNA encoded by nuclear genes (Table 1). This protein not only initiates mtDNA transcription and replication but also maintains mtDNA structure. Interestingly, the role of TFAM in mitochondria is similar to the role of histones in the nucleosome. TFAM wraps mtDNA entirely to form a nucleoid structure that may protect mtDNA against ROS. TFAM influences the tumor microenvironment by affecting the ratio of cytotoxic T cells to T helper cells, and the production of TNF-α, IL-6, IL-1β, and IL-12 p40 in DCs [80].

Cardiolipin (CL) is a phospholipid that accounts for 20% of the total lipid content in the inner mitochondrial membrane (Table 1). CL is composed of two phosphatidylglycerol backbones and a glycerol head group. Four fatty acid chains of varying lengths and saturation are bound to CL. This phospholipid is pivotal in many mitochondrial processes, including protein import, dynamics, respiratory chain functionality, and metabolism. Cellular necrosis exposes CL to the extracellular medium, where it can be sensed by T cells via antigen-presenting glycoprotein CD1d. In addition, CL can bind directly to NLRP3 and activate an inflammasome-mediated immune response [81].

Thus, mitochondria serve as a significant source of intracellular DAMPs. The best-characterized mtDAMPs, i.e., mtDNA, N-formyl peptides, TFAM, and cardiolipin, induce inflammatory responses through interaction with TLR9 (via MYD88/NF-κB), the NLRP3 inflammasome (leading to IL-1β production), and the cGAS-STING-IRF3 pathway. While autophagic recycling of damaged mitochondria normally prevents unwarranted inflammation, exceeding this homeostatic capacity or dysfunction of these protective systems promotes the development of autoimmune, infectious, and neoplastic diseases.

##### Nuclear DAMPs

DAMPs of nuclear origin (nDAMPs) include HMGB1 and nucleosomal components, such as DNA and histones. In addition to acting individually, these nDAMPs are frequently present in complexes that mediate inflammatory and immune responses [101].

Nuclear DAMPs are endogenous molecules that are normally confined to the cell nucleus and perform essential regulatory functions. Their interaction with PRRs activates signaling cascades that lead to the production of proinflammatory cytokines, including IL-1β, TNF-α, and IFN-γ. These cytokines recruit immune cells, such as macrophages and neutrophils, to the site of injury and also promote the development of adaptive immune responses by participating in antigen presentation [102].

The principal representatives of nuclear DAMPs are listed in Table 1.

Nuclear DNA, normally localized within the nucleus, can be released into the cytoplasm or extracellular space when cellular membrane integrity is compromised. Single-stranded DNA containing CpG motifs can activate TLR9, thereby inducing a proinflammatory response.

Circulating free DNA (cfDNA) is thought to originate from apoptotic or necrotic cells and may therefore reflect systemic inflammatory processes or tissue damage [103,104,105].

Histone proteins are involved in the packaging of DNA into chromatin and carry a high positive charge, enabling efficient binding to negatively charged DNA. During necrotic cell lysis, histones can be released together with DNA fragments, forming nucleosomes that subsequently become accessible to the immune system. The interaction of histones with TLR4 induces an inflammatory response similar to that elicited by bacterial lipopolysaccharides (LPS). Nucleosomes can also be released and initiate inflammatory processes through interaction with innate immune receptors [85].

HMGB1 is a non-histone DNA-binding protein that plays an important role in the regulation of transcription and in maintaining chromatin architecture. Upon exposure to stress or cellular injury, HMGB1 can translocate from the nucleus to the cytoplasm and subsequently into the extracellular space, where it functions as a potent proinflammatory mediator. HMGB1 interacts with receptors such as RAGE and TLR4, thereby promoting activation of inflammatory signaling pathways [83,106].

Intracellularly, HMGB1 interacts with tumor protein p53, T-box transcription factor (TBR), octamer-binding transcription factor 4 (Oct4), homeobox (Hox) proteins, steroid receptors, and numerous viral proteins, thereby regulating gene expression [107]. HMGB1 has been shown to shuttle between the nucleus and cytoplasm depending on the phase of the cell cycle. In lymphoid cells, HMGB1 is present in both compartments. The presence of HMGB1 in the extracellular space is considered a marker of acute injury or necrosis, as it reflects irreversible chromatin damage. At sites of mechanical injury, HMGB1 interacts with RAGE, enhancing the production of TNF, IL-1, IL-8, monocyte chemoattractant protein-1 (MCP-1), stromal cell-derived factor 1α (SDF-1α), and other factors that recruit stem cells to the site of damage. HMGB1 can be released from cells both actively and passively. Active secretion is associated with its dissociation from chromatin following histone acetylation, HMGB1 hyperacetylation, and HMGB1 monomethylation. Passive release occurs during necrosis. In contrast, during normal non-immunogenic apoptosis, HMGB1 is not released from the tightly packed nuclei of apoptotic cells. Release of HMGB1 from necrotic tumor cells treated with doxorubicin at high concentrations, which induces necrosis, promotes tumor regrowth and metastasis via activation of the RAGE pathway [108].

Elevated HMGB1 levels in peripheral blood during the first days of epirubicin-docetaxel combination chemotherapy may indicate an early treatment response. A significant increase in HMGB1 levels was observed only in breast cancer patients who subsequently demonstrated tumor regression, whereas no changes were detected in non-responders [109]. Early increases in peripheral HMGB1 levels in response to chemotherapy have been shown to correlate with long-term survival in breast cancer [110], lung cancer [111], esophageal squamous cell carcinoma [112], and other malignancies [113]. Thus, HMGB1 may serve as a valuable additional biomarker for early prognostic assessment. However, no significant correlation has been observed between HMGB1 levels and tumor response after neoadjuvant chemotherapy or overall survival [114]. Furthermore, another study reported no significant prognostic value of HMGB1 in melanoma [115].

Thus, nuclear DAMPs play a critical role in the initiation and maintenance of inflammatory responses, coordinating host defense mechanisms in response to injury and infection.

##### Cytosolic DAMPs

Cytosolic DAMPs are molecules normally localized within the cytoplasm that, under certain conditions, can be released into the extracellular environment and activate immune responses (Table 1).

Calreticulin (CRT)

**Figure 4:**
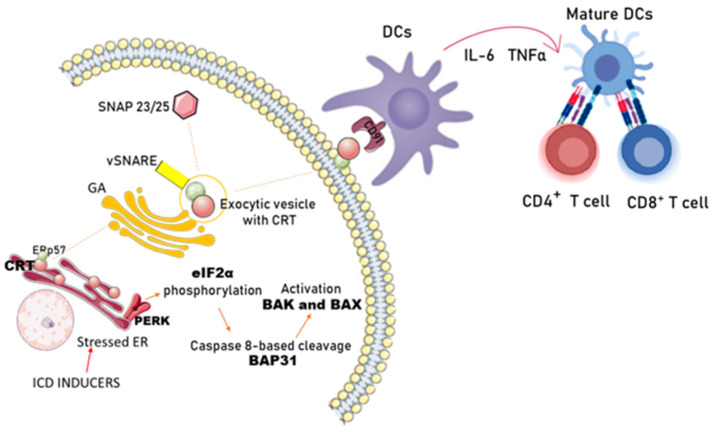
Exposure of calreticulin (CRT) on the outer plasma membrane and its recognition by dendritic cells. Stress factors that induce ICD disrupt ER homeostasis by activating unfolded protein response signaling pathways. During ICD, PERK activation leads to phosphorylation of eIF2α, which in turn promotes ER stress, pre-apoptotic cleavage of BAP31 by caspase-8, and activation of Bax/Bak. Subsequently, CRT translocates to the GA and is exocytosed via the SNARE machinery, fusing with the plasma membrane through interactions with SNAP23/25. This results in CRT exposure on the outer membrane of the dying cell. Surface-exposed CRT binds to CD91 on antigen-presenting cells (e.g., DCs), stimulating production of IL-6 and TNF-α and modulating Th1/Th17 cell activity. By promoting phagocytosis of dying cells, CRT recruits antigen-presenting cells to the tumor bed, thereby facilitating antigen presentation and the immune response. Reduced expression of CD47—an inhibitor of phagocytosis—enhances the immunostimulatory effects of CRT. Expression levels of CRT and/or CD47 correlate with disease outcome. Abbreviations: APC—antigen-presenting cells, Bak—BCL2-antagonist/killer, BAP31—B-cell receptor-associated protein 31, Bax—BCL2-associated X protein, CRT—calreticulin, DCs—dendritic cells, eIF2α—eukaryotic translation initiation factor 2 alpha, ER—endoplasmic reticulum, GA—Golgi apparatus, ICD—immunogenic cell death, PERK—RNA-like endoplasmic reticulum kinase, SNAP23/25—synaptosomal-associated protein 23/2, SNARE—soluble N-ethylmaleimide-sensitive factor attachment protein receptor, Th—T helper, TNF-α—tumor necrosis factor alpha.

CRT is a soluble protein of the ER, traditionally regarded as a regulator of Ca^2+^ homeostasis. It is involved in multiple functions both within and outside the ER, including regulation of chaperone activity, assembly of major histocompatibility complex class I (MHC I) molecules, and control of cell proliferation and migration. Certain anticancer strategies have been shown to induce ICD in tumor cells, characterized by the exposure of CRT on the cell surface prior to the onset of apoptosis. Surface-exposed calreticulin promotes tumor antigen presentation and tumor-specific cytotoxic T-lymphocyte responses [116]. In response to ICD inducers, CRT is translocated to the surface of cancer cells before phosphatidylserine exposure (Figure 4).

This process is independent of nuclear components, DNA damage, and transcriptional reprogramming. Anthracycline-induced CRT exposure requires the generation of reactive oxygen species and nitric oxide, as well as activation of the ER stress response, including phosphorylation of eukaryotic translation initiation factor 2 alpha (eIF2α) by protein kinase RNA-like ER kinase (PERK). This is typically followed by caspase-8-mediated proteolysis of the cytoplasmic domain of B-cell receptor-associated protein 31 (BAP31), activation of the proapoptotic proteins BCL2-associated X protein (BAX) and BCL2-antagonist/killer (BAK), anterograde transport of CRT from the ER to the Golgi apparatus, and exocytosis of CRT-containing vesicles, ultimately resulting in soluble N-ethylmaleimide-sensitive factor attachment protein receptor (SNARE)-dependent translocation of CRT to the plasma membrane. Disruption of this complex pathway at any level (by pharmacological or genetic interventions) abolishes CRT exposure, eliminates the immunogenicity of apoptosis, and reduces the immune response induced by anticancer chemotherapy [117,118].

Surface-exposed CRT binds to the CD91 receptor on macrophages and DCs. This interaction stimulates DC production of cytokines, such as IL-6 and TNF-α, which in turn modulate the activity of immunostimulatory T helper type 1 (Th1) cells and IL-17-producing T cells (Th17). Importantly, CRT acts as a potent pro-phagocytic signal on the surface of dying cells, promoting the recruitment of antigen-presenting cells (e.g., DCs) to the tumor bed, the engulfment of dead cells and cellular debris, efficient antigen processing, optimal presentation to T cells, and initiation of an appropriate immune response [8,23,119]. Notably, the immunostimulatory effects of cell surface CRT are strongly inhibited by co-expression of CD47, a phagocytosis-inhibitory signal expressed by various solid and hematopoietic tumors [120,121]. Expression levels of CRT and/or CD47 correlate with disease outcomes [121].

Studies indicate that elevated CRT protein levels may be useful for cancer diagnostics. For example, in patients with neuroblastoma, high CRT expression correlates with improved tumor differentiation and enhanced patient survival [122]. Elevated CRT levels have also been detected in the urine of patients with bladder carcinoma, suggesting its potential as a diagnostic marker [123]. Studies in patients with non-small cell lung cancer and acute myeloid leukemia have likewise demonstrated a beneficial association between high CRT levels and survival [124,125]. However, in patients with gastric cancer, elevated CRT expression has been associated with poor prognosis and an increased risk of death [126]. These conflicting findings underscore the need for further research to elucidate the role of CRT across different cancer types.

S100 proteins

Ca^2+^-binding proteins S100A8 and S100A9 belong to the calmodulin-like protein family and play an important role in the regulation of inflammatory processes (Table 1). They are typically localized within phagocytes but can be released in response to cellular stress or activation of protein kinases, such as protein kinase C. Upon release, these proteins function as DAMPs, promoting the induction of inflammation and fibrosis [127]. S100A9 promotes M1 polarization of macrophages [128]. Forming a common heterodimer structure S100A8/A9, S100A8 and S100A9 are widely reported to participate in multiple signaling pathways in tumor cells. Meanwhile, S100A8/A9, S100A8, and S100A9, mainly act as promoters, contributing to tumor development, growth and metastasis by interfering with tumor metabolism and the microenvironment [91].

Uric acid

Uric acid is a product of purine metabolism and is constitutively present in all cells. Its levels increase following cellular injury, making it an important endogenous DAMP (Table 1). Dying cells release uric acid into the extracellular space, where it can trigger immune responses. Activation of innate immunity involves cells such as macrophages, monocytes, NK cells, and neutrophils, leading to the secretion of proinflammatory cytokines or activation of the NACHT, leucine-rich repeat (LRR), and NLRP3 inflammasome, thereby amplifying inflammatory responses [86,87]. In a hyperuricemic environment, the number of classical monocytes increases, whereas the number of intermediate monocytes decreases. Serum uric acid (sUA) and monosodium urate (MSU) act on monocytes through multiple signaling pathways, such as NF-κB/NLRP3/GSDMD and solute carrier family 2 member 9/glucose transporter type 9 (SLC2A9/GLUT9), which ultimately affect the expression of inflammatory factors [88]. Uric acid induces dysregulation of macrophage glucose metabolism, leading to inflammation. Uric acid crystals activate macrophage glycolysis, thereby enhancing the expression of inflammatory factors [89]. Alternatively, adaptive immunity may be induced either through activation of innate immunity or via direct effects on T cells [129]. Various studies have demonstrated that sUA or MSU crystals exert regulatory effects on CD8^+^ T cells, including their proliferation [90], recruitment [130], and polarization [131]. These effects are implicated in a range of inflammatory and autoimmune diseases, including gout [88,132].

Heat shock proteins (HSPs)

HSPs are a family of proteins that typically function as molecular chaperones, facilitating proper protein folding and preventing the aggregation of misfolded proteins. However, under conditions such as cellular stress, apoptosis, HSPs—primarily HSP70 and HSP90—can be released into the extracellular space and act as DAMPs (Table 1). They are capable of interacting with toll-like receptors, such as TLR4, thereby activating inflammatory signaling pathways [133,134]. HSP70- and HSP90-mediated activation of low-density lipoprotein receptor-related protein 1 (LRP1) can promote cancer cell metastasis [135,136].

Adenosine triphosphate (ATP)

ATP release may occur passively, for example during necrosis; however, multiple molecular pathways for active release have been described. These include exocytosis of ATP-containing lysosomes in astrocytes, pannexin-mediated ATP release during apoptosis, and connexin- or pannexin-mediated ATP release in inflammatory cells such as neutrophils [31]. Furthermore, recent studies have demonstrated that ATP can also be released by dying cancer cells via the classical endoplasmic reticulum/Golgi secretory pathway [93,137]. In the extracellular compartment, nucleotide signaling is inherently short-lived. Signaling terminates within seconds to minutes due to the enzymatic conversion of ATP to adenosine by ectonucleoside triphosphate diphosphohydrolase CD39 (converting ATP/ADP to AMP) and ecto-5′-nucleotidase CD73 (converting AMP to adenosine) [138]. ATP functions as a signaling molecule through activation of P2 purinergic receptors. These receptors are widely expressed across various tissues and are involved in both innate and adaptive immune responses. P2 receptors are classified into metabotropic P2Y receptors (P2YRs), which are G protein-coupled, and ionotropic P2X receptors (P2XRs), which are ligand-gated ion channels [139]. P2YR signaling is associated with chronic inflammation, and one of the most extensively studied receptors in this class is P2Y2R, which is activated by UTP or ATP. P2Y2R agonists promote mucociliary clearance and wound healing and have therefore been used in the treatment of cystic fibrosis. Apoptotic cells release ATP as a “find-me” signal, which binds to P2Y2R on the surface of macrophages, stimulating their phagocytic activity and facilitating the clearance of apoptotic cells [93].

In summary, the key representatives of cytosolic DAMPs we examine, including ATP, CRT, HSPs, S100 proteins, and uric acid, play a critical role in inducing and modulating immune responses upon release into the extracellular environment during cellular stress or following cell death. Each of these DAMP ligands engages specific receptors and signaling pathways, leading to the recruitment of antigen-presenting cells, production of pro-inflammatory cytokines, activation of phagocytosis, and enhancement of antitumor immunity. However, the effects of these molecules can be bidirectional: they not only promote tumor cell elimination (e.g., CRT and ATP) but may also, depending on the microenvironment and pathology type, act as promoters of inflammation, fibrosis, metastasis, or poor prognosis. Thus, cytosolic DAMPs represent important mediators of intercellular signaling, and their regulation is a promising target for therapeutic intervention, requiring further context-dependent investigation.

## 4. Discussion and Future Perspectives

To date, a considerable number of DAMPs have been identified, and their list continues to expand. DAMPs play a crucial role in activating antitumor immune responses and ultimately influence disease outcomes. DAMPs serve as the main hallmarks of ICD. Emitted from dying cells, DAMPs act as adjuvants for antigen-presenting cells (such as macrophages and DCs), facilitating their recruitment to the tumor site and promoting their phagocytic activity and maturation. In combination with the antigenicity of the ICD mechanism [7,8], the subsequent development of a sustained antitumor response serves as a major driving force for the effective control of tumor growth and long-term anticancer immunity. Therefore, developing antitumor strategies that activate ICD is considered a highly promising approach for effective cancer immunotherapy.

It should be emphasized that while all ICD modalities lead to the release of DAMPs, the composition (Table 1), kinetics, and quantity of DAMP release, i.e., the ICD-associated DAMP profile, vary significantly depending on the specific ICD inducer and the cell type. This directly affects the efficiency of ICD activation and the subsequent development of a sustained antitumor immune response. Moreover, some aggressive tumor types, such as glioblastoma, can acquire resistance to therapies based on the induction of classical RCD modalities like apoptosis [140] or have inherent resistance to necroptosis [141]. A promising strategy to overcome resistance is the activation of alternative RCD classified as ICD, such as ferroptosis [142], or, even better, the use of a multimodal approach inducing mixed-type cell death with immunogenic properties [143]. Ensuring the switch from one cell death mechanism to an alternative pathway would enable targeting of the entire heterogeneous tumor population, preventing the activation of adaptive mechanisms and the development of resistance—the latter often occurring with monotherapy. Furthermore, ICD activation would also help form an immunologically “hot” tumor microenvironment [144,145,146], thereby facilitating the further development of the antitumor immune response.

However, several challenges must be considered when developing an effective therapeutic strategy based on ICD induction. Although DAMPs are key hallmarks of ICD, their release is not always accompanied by ICD activation. Some molecules currently classified as DAMPs may also be released during non-specific cell death, such as necrosis or accidental cell injury [24,32,147]. Additionally, the blockade of DAMP release through proteome regulation by tumor cells [121], secretion of mutant DAMPs [148], or the action of pharmacological agents [119] can disrupt the dialog between dying tumor cells and the immune system, thereby reducing ICD efficacy. Moreover, the tumor microenvironment is not a passive bystander but an active, multilayered saboteur of DAMP-mediated immunity, targeting the release, stability, recognition, and downstream signaling of danger signals to establish immune privilege. The tumor microenvironment can promote autophagy, thereby disrupting the release of several key DAMPs, primarily ATP, and consequently, ICD mechanisms [149,150]. The tumor microenvironment can also neutralize the effects of DAMPs already released. For example, ATP can be converted into immunosuppressive adenosine via CD39/CD73 in the tumor microenvironment [138,151]. Furthermore, oxidation of HMGB1 by ROS can occur in the microenvironment. The oxidized form of HMGB1 loses its ability to activate TLR4 and may promote the development of immune tolerance [107,152]. The tumor microenvironment can also trigger desensitization of key receptors (TLR4, P2X7, cGAS-STING) on infiltrating antigen-presenting cells, thereby suppressing their phagocytic activity in the presence of DAMPs and subsequent maturation [153,154,155].

Thus, despite their favorable effects, DAMPs can also exert pro-tumor effects, thereby contributing to tumor progression (Table 2). Therefore, for each tumor type, an appropriate bona fide ICD inducer and its treatment regimen should be carefully selected.

Measuring DAMP levels can serve as both diagnostic and prognostic biomarkers, significantly complementing the clinical assessment of anticancer therapy efficacy. In line with this, the greatest interest has focused on HMGB1 (a nuclear DAMP) [185,186,187] and CRT (a cytosolic protein) [118,122,164,188] as the most significant markers identified to date. Their detection in tumor biopsies, surgically resected tissue samples, or peripheral blood using immunohistochemical analysis and/or ELISA allows for the prediction of disease course and assessment of the risks of tumor progression. However, it should be noted that the dynamics of detectable DAMP levels and the interpretation of results vary greatly depending on tumor type. Therefore, these markers must be interpreted in a context-dependent manner [19,187,189].

An equally important aspect of translating DAMPs into clinical practice is their use in developing novel antitumor therapies. Special attention is given to DAMPs as adjuvants for anticancer vaccines, including those designed to promote ICD induction [190] and DAMP-enriched vaccines (e.g., DPV-001 vaccine [191,192,193]). Another promising approach that addresses multiple therapeutic goals is the development of new nanomaterials for targeted delivery of antitumor agents combined with ICD inducers, as demonstrated in comprehensive preclinical studies [194]. Nanomaterials enable rational combinations of therapeutic agents that amplify protective immunity while extinguishing chronic DAMP-driven inflammation, thereby avoiding adverse cytotoxic and protumorigenic effects.

Therefore, DAMP-based cancer therapy stands at a translational crossroads. Monitoring DAMPs and the processes associated with their release may have dual clinical significance. First, it may improve patient stratification by identifying individuals with different prognoses and those likely to respond to specific therapeutic regimens. Second, it may facilitate therapy selection by identifying molecular or cellular defects specific to individual patients. While the immunogenic potential of DAMPs is undeniable, harnessing their power requires moving beyond monotherapy approaches toward intelligent, context-aware systems. Future development of such immunotherapeutic strategies may enable the modulation of therapeutic outcomes using targeted pharmacological interventions.

## 5. Conclusions

This review summarizes the current understanding of DAMPs as active inducers of antitumor immunity. Despite growing knowledge about this type of molecular signal, the nuclear protein HMGB1 and the endoplasmic reticulum protein CRT remain the most potent DAMPs. The classical concept of ICD links it to various mechanisms that activate T-cell-dependent specific immune responses, with HMGB1 and CRT being the strongest inducers. However, mutations in these proteins or oxidative structural modifications caused by carcinogenesis can disrupt the mechanisms underlying the generation of immune responses and, in some cases, even promote tumor progression and metastasis. Thus, exploring the molecular mechanisms of ICD activation and the specific release profiles of DAMPs is a highly promising avenue for developing novel anticancer treatment strategies.

## Figures and Tables

**Table 1:** Key DAMPs and pathways of immune response activation.

Name	Cause of Release	Effect	Receptor	References
**Extracellular DAMPs**				
Proteoglycans and glycoproteins	Extracellular matrix degradation	Fibroblast activation; inflammation and fibrosis; induction of IL-6, IL-8, and TNF-α release	PRR	[73,74,75,76]
Tenascin C	Pathological alternative mRNA splicing		TLR4	
Aggrecan, 32-mer fragment	MMP, ADAMTS cleavage		TLR2, and NF-κB-dependent signaling	
Biglycan	MMP cleavage, de novo synthesis		TLR2, TLR4, NLRP3	
Decorin	MMP cleavage, de novo synthesis		TLR2, TLR4	
Fibronectin	MMP, alternative splicing, unfolding		TLR2, TLR4	
Fibrinogen	Extravasation		TLR4	
Versican	Secretion		TLR2, TLR6, CD14	
**Mitochondrial DAMPs**				
Mitochondrial DNA (mtDNA)	Mitochondrial membrane permeability disruption	cGAS/STING/IRF3	TLR9	[79]
Mitochondrial transcription factor A (TFAM)	Mitochondrial damage; apoptosis; cleaved from mtDNA	cGAS-STING pathway activation in DCs; production of TNF-α, IL-6, IL-1β and IL-12 p40	RAGE	[80]
N formyl peptides (NFP)	Mitochondrial DNA degradation	Neutrophil chemoattraction	FPR	[81]
Cardiolipin	Necrosis, necroptosis	NLRP3 activation	CD1d	[81,82]
**Nuclear DAMPs**				
High-mobility group box 1 (HMGB1)	Translocation to cytoplasm and extracellular space	Production of TNF, IL-1, IL-8, MCP-1, SDF-1α; release during necrosis—activation of metastasis via RAGE	RAGE, TLR4	[83]
DNA (cfDNA, single-stranded DNA)	Loss of membrane integrity	Systemic inflammatory response	TLR9	[84]
Histones	Necrosis, apoptosis, NETosis	CXCL9, CXCL10, IL1β, IL6, IL8, IL18, TNF-α, IFN-α, leukocyte adhesion and migration	TLR4	[85]
**Cytosolic DAMPs**				
Calreticulin (CRT)	Endoplasmic reticulum dysfunction; early exposure on the cell surface	Activation of DCs and macrophages, IL-6 and TNF-α induction, Th1, IL-17, Th17 activation, increased CD47 co-expression, inhibition of the effect	CD91	[19]
Uric acid	Increased levels after cellular damage	Activation of the NACHT inflammasome, LRR and NLRP3. As a consequence, activation of monocytes, NK cells and neutrophils, activation of glycolysis in macrophages, CD8^+^ T-cell proliferation	NF-κB/NLRP3/GSDMD and SLC2A9/GLUT9	[86,87,88,89,90]
S100A8 and S100A9	Release from phagocytes; protein kinase C activation	Induction of inflammation and fibrosis, promotion of M1 macrophage polarization	TLR4, RAGE	[91]
Heat shock proteins (HSPs)	Apoptosis, necrosis	TLR4—inflammation; LRP1—metastasis	TLR4 LRP1	[92]
Adenosine triphosphate (ATP)	Passive release (necrosis); during ICD: lysosomes, pannexin- and connexin-dependent mechanisms, via the Golgi apparatus	Stimulation of macrophage phagocytosis	P2 (P2YR, P2XR)	[93]

Abbreviations: ADAMTS—a disintegrin and metalloproteinase with thrombospondin motifs, ATP—adenosine triphosphate, cfDNA—circulating free DNA, cGAS—cyclic GMP-AMP synthase, CRT—Calreticulin, CD1d—antigen-presenting glycoprotein, CD—cluster of differentiation, CXCL—C-X-C motif chemokine ligand, DCs—dendritic cells, FPR—formyl peptide receptor, GLUT9—glucose transporter type 9, GSDMD—gasdermin D, HMGB1—high-mobility group box 1, HSPs—heat shock proteins, ICD—immunogenic cell death, IL—interleukin, IRF3—interferon regulatory factor 3, LRR—leucine-rich repeat, LRP1—low-density lipoprotein receptor-related protein 1, MMP—matrix metalloproteinase, mtDNA—mitochondrial DNA; MCP-1—monocyte chemoattractant protein-1, NETosis—neutrophil extracellular trap formation, NF-κB—nuclear factor kappa-light-chain-enhancer of activated B cells, NK—natural killer, NLRP3—NLR family pyrin domain containing 3, PRR—pattern recognition receptors, P2XR—purinergic receptor P2X, P2YR—purinergic receptor P2Y, RAGE—receptor for advanced glycation end-products, SDF-1α—stromal cell-derived factor 1α, SLC2A9—solute carrier family 2 member 9, STING—stimulator of interferon genes, TFAM—mitochondrial transcription factor A, Th1—T helper 1, Th17—T helper 17, TNF-α—tumor necrosis factor alfa, TLR—toll-like receptor.

**Table 2:** Pros and cons: the role of major DAMPs in tumorigenesis.

DAMP	Pro-Tumorigenic Mechanism	Anti-Tumorigenic Mechanism	References
Adenosine triphosphate (ATP)	**Immunosuppression;** **Proliferation and migration:** activation of the purinergic receptor P2X7 leads to the release of vesicles containing the ectonucleotidases CD39 and CD73, which increase ATP levels; **Maintenance of stemness** via the PI3K/AKT kinase pathway; **Acquisition of drug resistance.**	**Induction of immunogenic cell death:** release of ATP through PANX1 channels acts as a key “find-me” signal, attracting dendritic cells (DCs). In parallel, TNFα promotes PANX1 cleavage through the caspase-8/3-dependent pathway, thereby enhancing ATP release. DCs are then activated via P2X7 and P2Y2 receptors, leading to their maturation, migration to lymph nodes, and presentation of tumor antigens to T cells. **Antigen presentation and TIL recruitment:** engulfment of dying cells, cross-presentation of tumor antigens to T cells, and recruitment of tumor-infiltrating lymphocytes occur via the NF-κB pathway, RIPK-dependent NLRP3 inflammasome signaling, and PANX1-mediated signaling downstream of caspase-3 cleavage; **ATP release and leukocyte recruitment:** in response to anticancer therapeutic agents, ATP release through caspase-3-driven PANX1 channels enhances leukocyte recruitment and promotes dendritic cell motility via the P2X7 receptor upon sensing danger signals.	[156,157,158,159,160]
Calreticulin (CRT)	**Increased proliferative activity and survival**: activation of NF-κB, JAK2, FAK/ERK/MAPK, and STAT5 leads to tumor progression; **Enhanced expression of oncogenic factors**: EIF2AK2, MMP14, ADAR1 et al.; **Metastasis:** interaction with methyltransferase G9a induces hypermethylation of the E-cadherin promoter, disrupts the regulation of embryonic transformation markers (cadherins, ZO-1, Snail, ZEB1, etc.) and adhesion molecules (fibronectin, integrin β1, MMP2); **Neovascularization:** stabilization of mRNA of proangiogenic factors PIGF and VEGF; **Immunosuppression:** activation of the ERK/Sp1/TGFβ1 axis, leading to Treg proliferation and suppression of T-cell cytotoxicity.	**Induction of immunogenic cell death:** exposure of CRT on the outer surface of the plasma membrane serves as an “eat me” signal promoting activation of phagocytosis of antigen-presenting cells via interaction with LRP1 (CD91); **Dendritic cell activation:** interaction with TLR4 on DCs activates the MyD88 signaling pathway and increases secretion of TNFα and CCL19, which attract and activates DCs; **Macrophage activation via LRP1:** the p38 MAPK and NF-κB pathways trigger the synthesis and release of pro-inflammatory cytokines such as TNF-α, IL-6, and IL-1β; **Suppression of the Wnt/β-catenin** signaling pathway: this reduces tumor growth.	[161,162,163,164,165,166]
High-mobility group box 1 (HMGB1)	**Neoangiogenesis:** the NF-κB, HIF-1α, and JNK pathways enhance the expression of vascular endothelial growth factor (VEGF). **Activation of autophagy** via the MEK/ERK signaling pathway; **Epithelial–mesenchymal transition** via the RAGE/NF-κB, TLR2/PI3K/Akt, MAPK, and PI3K (via TLR4) signaling pathways; **Extracellular matrix degradation** via the NF-κB and TLR4/MyD88 signaling pathways, and invasion via MMPs; **Stimulation of chronic inflammation:** the NF-κB signaling pathway leads to the production of pro-inflammatory cytokines (TNF-α, IL-6) and activation of the NLRP3 inflammasome; **Inhibition of apoptosis:** the PI3K/Akt and MAPK/ERK signaling pathways promote suppression of apoptosis by increasing Bcl-2 levels and decreasing Bax levels; **Enhancement of proliferative activity:** increased levels of the protooncogenes cyclin D1 and c-Myc.	**Induction of immunogenic cell death modalities:** increased reactive oxygen species (ROS) levels via the RAS-JNK/p38 signaling pathway; **Macrophage polarization toward an antitumor M1 phenotype**: direct or via the ROS/HMGB1 or HMGB1/TLR4/NLRP3 pathways; **Activation of antitumor immunity:** TLR4 on DCs promotes tumor antigen presentation and naïve T cells activation, thereby enhancing the antitumor response; **Induction of tumor cell senescence:** p53-dependent irreversible cell cycle arrest; **Inhibition of angiogenesis:** blockade of the RAGE/MEK/ERK signaling pathway.	[107,167,168]
Heat shock proteins (HSPs)	**Resistance to apoptosis**: inhibition of tumorous imaginal disk (Tid1) and p53, clusterin, and cyclophilin D **Stemness** of tumor cells; **Reduction in surface expression** of CD3, CD4, CD8, CD28, CD40L, CD25, and αβ on T-cells, as well as activating receptors (CD2, CD11a, CD94, NKp30, NKp44, NKp46, KARp50.3) on NK-cells; **Angiogenesis**: stabilization of HIF-1α stimulates the production of VEGF and other pro-angiogenic factors; **Migration, invasion, and metastasis**: increased VEGF and elevated TGF-β activate HSPs, interact with MMP2, and promote EMT; **Arrest of cellular senescence** by inhibiting p53 and p21 production and activating PTEN; **Maintenance of proliferative activity and tumor cell survival.**	**Enhance** the cytotoxicity of NK cells, promote the maturation of dendritic cells (via TLR2/TLR4 receptors), and increase cytokine secretion by monocytes; **Induce** a strong CD4+ and CD8+ T-cell response and enhance the immunosuppressive activity of myeloid-derived suppressor cells and regulatory T cells; **Suppress** Th1-associated transcription factors (i.e., T-bet, NF-kB, NFATp) and activate GATA3, thereby reducing TNF-α and IFN-γ secretion while increasing IL-10, IL-4, and IL-13.	[169,170,171,172]
Mitochondrial DNA (mtDNA)	**Immunosuppression**: the STING protein, acting via the cGAS-STING-NF-κB signaling pathway, activates type I interferon and NF-κB signaling. This leads to increased production of Arg1, Nos2, and Cd247 (PD-L1) in myeloid-derived suppressor cells, which exert a potent inhibitory effect on T-cells; **T-cell dysfunction:** transfer of mitochondria with mtDNA mutations from cancer cells to T-cells in the tumor microenvironment leads to T-cell dysfunction; **M2 polarization of macrophages:** binding to TLR9 on macrophages in the tumor microenvironment promotes their polarization into an immunosuppressive M2 phenotype; **Metastasis:** enhanced oxidative phosphorylation in mitochondria drives metastasis; **Development and progression**: caused by oncogenic mutations and mtDNA damage.	**Induction of immunogenic cell death modalities:** (by activating cGAS-STING signals); Activation of the NLRP3 inflammasome, IL-1β and IL-18; IFN-I signals activate dendritic cells and promote the maturation and recruitment of cytotoxic CD8+ T lymphocytes; TLR9 is recruited and activated by MyD88, which then activates IRAK-4, IRAK-1, TRAF6, and NF-κB, leading to enhanced transcription of inflammatory cytokines; The oxidized form of mitochondrial DNA (ox-mtDNA) acts as a potent molecular signal that triggers the activation of pattern recognition receptors and enhances sterile inflammation.	[173,174,175,176,177]
Circulating free DNA (cfDNA)	**Metastasis:** elevated levels of cfDNA in the blood correlate with the presence of lymph node metastases; **Immunosuppression:** direct interaction of cfDNA with major histocompatibility complex class II (MHC II) molecules on antigen-presenting cells leads to recruitment of regulatory T-cells; **Drug resistance:** high levels of cfDNA in the blood serve as an independent prognostic parameter for poor response to immunotherapy and chemoradiotherapy.	Activation of the cGAS-STING signaling pathway triggers the production of pro-inflammatory type I interferon (IFN-I); IFN-I signals subsequently activate DCs, promoting the maturation and recruitment of cytotoxic CD8+ T lymphocytes.	[103,178,179,180,181]
Proteoglycans	**Angiogenesis:** influence on blood vessel development via glycosaminoglycan chains and interaction with angiogenic factors (e.g., heparan sulfate proteoglycans (HSPGs) stabilize VEGF and FGF, thereby enhancing neovascularization); **Participation in invasion and metastasis:** versican and aggrecan promote basement membrane destruction via CD44/MMP-9; **Activation of autophagy:** some proteoglycans (e.g., syndecan-1) regulate autophagy as a survival mechanism under stress conditions; **Resistance to therapy:** fibromodulin, biglycan, and other proteoglycans activate survival-related signaling pathways (EGFR, PI3K/AKT, NF-κB, ERK), reducing sensitivity to apoptosis and diminishing the efficacy chemotherapy and targeted therapy; **Immunosuppression:** proteoglycans in the tumor microenvironment modulate the activity of tumor-associated macrophages (TAMs) and myeloid-derived suppressor cells (MDSCs).	**Suppression of tumor growth and proliferation:** small leucine-rich proteoglycans (decorin, biglycan, lumican) inhibit proliferation by binding to EGFR and other receptor tyrosine kinases; **Inhibition of metastasis and angiogenesis**: decorin suppresses MMP expression and activates pathways that stabilize E-cadherin. Additionally, decorin and biglycan bind VEGF, block its interaction with receptors, and induce the production of endogenous inhibitors such as thrombospondin-1.	[182,183,184]

Abbreviations: ADAR1—adenosine deaminase acting on RNA 1, AKT—AKT serine/threonine kinase (protein kinase B), Bax—Bcl-2-associated X protein, Bcl-2—B-cell lymphoma 2, CD—cluster of differentiation, cfDNA—circulating free DNA, cGAS-STING—cyclic GMP-AMP synthase, DC—dendritic cells, EGFR—epidermal growth factor receptor, EIF2AK2—eukaryotic translation initiation factor 2-alpha kinase 2, ERK—extracellular signal-regulated kinase, FAK—focal adhesion kinase, FGF—fibroblast growth factor, IRAK—interleukin-1 receptor-associated kinase, JAK2—Janus kinase 2, JNK—C-Jun N-terminal kinase, LRP1—low-density lipoprotein receptor-related protein 1, MAPK—mitogen-activated protein kinase, MDSC—myeloid-derived suppressor cells, MEK—mitogen-activated protein kinase, MHC—major histocompatibility complex, MMP—matrix metalloproteinase, MYD88—myeloid differentiation primary response gene (88), NC—neural crest cells, NFAT—nuclear factor of activated T cells, NF-κB—nuclear factor kappa-light-chain-enhancer of activated B cells, NLRP3—NOD-like receptor protein 3, PANX1—pannexin 1, PD-L1—programmed death-ligand 1, PI3K—phosphoinositide 3-kinase, PIGF—placental growth factor, PTEN—phosphatase and tensin homolog deleted on chromosome 10, RAGE—receptor for advanced glycation end-products, RAS—retrovirus associated DNA, RIPKs—receptor-interacting protein kinases, ROS—reactive oxygen species, Sp—serine protease, STAT5—signal transducer and activator of transcription 5, TAM—tumor-associated macrophages, TGF-beta—transforming growth factor beta, TNFα—tumor necrosis factor-alpha, VEGF—vascular endothelial growth factor, Wnt—wingless-type MMTV integration site family, ZEB1—zinc finger E-box binding homeobox 1, ZO-1—zonula occludens-1.

## Data Availability

No new data were created. Data sharing is not applicable to this article.

## Abbreviations

The following abbreviations are used in this manuscript:

Aβ	Beta-amyloid
ADAMTS13	A disintegrin and metalloproteinase with a thrombospondin type 1 motif, member 13
AKT	AKT serine/threonine kinase (protein kinase B)
ANXA1	Annexin A1
ASC	Apoptosis-associated speck-like protein containing a CARD
ATF6	Activating transcription factor 6
ATP	Adenosine triphosphate
Bak	BCL2-antagonist/killer
BAP	BRCA1-associated protein 1
Bax	BCL2-associated X protein
Casp	Caspase
CCL2	C-C motif chemokine ligand 2
CD40	Cluster of differentiation 40
cfDNA	Circulating free DNA
cGAS	Cyclic GMP-AMP synthase
CHOL	Cholesterol
CL	Cardiolipin
CLRs	C-type lectin receptors
CRT	Calreticulin
CTLs	Cytotoxic T lymphocytes
CXCL1	C-X-C motif chemokine ligand 1
CXCL10	C-X-C motif chemokine ligand 10
CPS1	Carbamoyl phosphate synthase 1
DAMPs	Damage-associated molecular patterns
DCs	Dendritic cells
eATP	Extracellular adenosine triphosphate
eCIRP	eCIRP—extracellular cold-inducible RNA-binding protein
eIF2α	Eukaryotic translation initiation factor 2 alpha
ER	Endoplasmic reticulum
ERK	Extracellular signal-regulated kinase
EVs	Extracellular vesicles
FasL/FasR	Fas ligand/Fas receptor
FPR	Formyl peptide receptor
GLUT	Glucose transporter
GSDMD	Gasdermin D
HMGB1	High-mobility group box 1
HSPs	Heat shock proteins
IAPP	Islet amyloid polypeptide
ICD	Immunogenic cell death
IFN (I)	Type (I) interferon
IL-6	Interleukin 6
IRE1α	Inositol-requiring enzyme 1 alpha
IRF3	Interferon regulatory factor 3
LPS	Lipopolysaccharides
LRP1	Low-density lipoprotein receptor-related protein 1
LRR	Leucine-rich repeat
MAPK	Mitogen-activated protein kinase
MBL	Mannose-binding lectin
MEK	Mitogen-activated protein kinase kinase (MAPK/ERK kinase)
MHC I/MHC II	Major histocompatibility complex class I/class II
MICA/B	MHC class I polypeptide-related sequence A/B
MMP	Matrix metalloproteinase
MSU	Monosodium urate
mtDNA	Mitochondrial DNA
mtDAMPs	Mitochondria-associated damage-associated molecular patterns
mtROS	Mitochondrial reactive oxygen species
MYD88	Myeloid differentiation primary response 88
NACHT	Domain present in NAIP, CIITA, HET-E, and TP1
NETosis	Neutrophil extracellular trap formation
NF-κB	Nuclear factor kappa-light-chain-enhancer of activated B cells
NFP	N formyl peptides
NK	Natural killer cells
NKG2D	Natural killer group 2 member D
NLRP3	NLR family pyrin domain containing 3
NLRs	NOD-like receptors
NMHC-IIA	Non-muscle myosin heavy chain IIA
NOD	Nucleotide-binding oligomerization domain
Oct4	Octamer-binding transcription factor 4
OSEs	Oxidative-specific epitopes
oxLDL	Oxidized low-density lipoprotein
p53	Tumor protein p53
P2RX2	Purinergic receptor P2X 2
P2RY7	Purinergic receptor P2X 7
PAK1	P21-activated kinase 1
PAMPs	Pathogen-associated molecular patterns
PERK	Protein kinase RNA-like endoplasmic reticulum kinase
PGs	Proteoglycans
PI3K	Phosphoinositide 3-kinase
PRRs	Pattern recognition receptors
PYD	Pyrin domain
Rac1	Rac family small gtpase 1
RAGE	Receptor for advanced glycation end products
RLRs	RIG-I-like receptors
RNA	Ribonucleic acid
ROS	Reactive oxygen species
SDF-1α	Stromal cell-derived factor 1α
SLC2A9	Solute carrier family 2 member 9
SNAP23/25	Synaptosomal-associated protein 23/25
SNARE	Soluble N-ethylmaleimide-sensitive factor attachment protein receptor
STING	Stimulator of interferon genes
sUA	Serum uric acid
TBR	T-box transcription factor
Th1	T helper 1
TFAM	Transcription factor A, mitochondrial
TLRs	Toll-like receptors
TNF-α	Tumor necrosis factor alpha
TXNIP	Thioredoxin-interacting protein
ULBPs	UL16-binding proteins
UPR	Unfolded protein response
